# Automated Digital Image Analysis (TrichoScan®) for Human Hair Growth Analysis: Ease versus Errors

**DOI:** 10.4103/0974-7753.66905

**Published:** 2010

**Authors:** Punit P Saraogi, Rachita S Dhurat

**Affiliations:** Department of Dermatology, T.N.M. College and B.Y.L. Nair Ch. Hospital, Mumbai Central, Mumbai, India

**Keywords:** Computer-assisted image analysis, digital image analysis, methodology, systematic errors, TrichoScan^®^

## Abstract

**Background:**

TrichoScan^®^ is considered to be time-saving, easy to perform and consistent for quantifying hair loss/growth. Conflicting results of our study lead us to closely observe the image analysis, and certain repeated errors in the detection of hair were highlighted.

**Aims:**

To assess the utility of TrichoScan in quantification of diffuse hair loss in males with androgenetic alopecia (AGA) and females with diffuse telogen hair loss, with regard to total hair density (THD), telogen and vellus hair percentages.

**Materials and Methods:**

TrichoScan procedure was performed on 77 cases and 20 controls.

**Results and Discussion:**

In the cases, THD decreased with increasing severity of alopecia. Surprisingly, more than 85% of the healthy volunteers had an unexplained abnormal telogen hair percentage of more than 20. Also, the telogen hair percentages were not significantly different between cases and controls. Also, 65% of the patients with advanced thinning of hair did not have the expected elevation of vellus hair percentages on TrichoScan evaluation. Multiple errors were highlighted in hair detection by the software. Errors were noted at the exit points of follicular ostia, at places where hair strand thickness was not uniform throughout its length, where there was crossing, overlapping of the neighboring strands, and when more than one hair emerged from a single ostium.

**Conclusion:**

TrichoScan is promoted as a validated and precise tool for measurement of hair growth parameters. Under certain conditions, it may seem suitable for clinical trials evaluating treatment response. We provide evidence that this is an overstatement. This study concludes that TrichoScan-analyzed anagen/telogen hair detection is not optimal; moreover, there is overestimation of THD and the vellus hair percentage does not correlate with clinical severity of alopecia. The current form of TrichoScan, though easy to use, is error-prone and awaits refinement.

## INTRODUCTION

Time and again, clinicians as well as researchers have tried to devise various methods for the analysis of human hair growth. Various methods available for quantification of hair loss/growth are classified as invasive (e.g., scalp biopsy), semi-invasive (e.g., trichogram, unit area trichogram) or noninvasive (e.g., global hair counts, phototrichogram (PTG), trichoscopy, TrichoScan^®^).

Non-invasive methods are preferred for long-term monitoring of patients of hair loss for objective quantification and to document response to therapy. PTG and contrast-enhanced phototrichogram (CE-PTG) involves manual identification of hairs and is considered as the most precise method of measurement, which has been validated with transverse sections of scalp biopsies.[1] However, CE-PTG is a tedious, time consuming process which is subject to human error.

Although various attempts at computer-assisted image analysis have been underway since 1980, the intrinsic complexity of hair growth has been the main impediment in successful automatization of the CE-PTG procedure.[2,3] However, in 2001, TrichoScan was launched as a fully-automated method for the measurement of biological parameters of hair growth such as hair density, diameter, anagen-telogen percentages as well as growth rate. According to the inventor, TrichoScan is investigator independent, very precise, reproducible, time-saving and easy to handle.[4]

Recently, in 2009, a study validated the TrichoScan technology and concluded that there is an excellent correlation between evaluation of hair parameters using manual identification of hairs and the fully-automated TrichoScan method. It further proposed that TrichoScan is particularly suitable for clinical studies with treatment comparisons.[5] Currently, TrichoScan is being used as a tool for quantifying hair loss across the globe in individual clinics, academic centers and clinical studies.

The aim of this case-control study was to assess the utility of TrichoScan in quantification of diffuse hair loss in females presenting with diffuse telogen hair loss of the scalp and males with androgenetic alopecia (AGA) by comparing TrichoScan-analyzed hair parameters, viz., total hair density (THD), telogen hair percentage and the vellus hair percentage, and to assess the relationship of clinical presentations of cases with TrichoScan-analyzed parameters.

## MATERIALS AND METHODS

The study was approved by the Institutional review board and written informed consent was taken from the participants of the study.

### Subjects

Thirty-seven female cases with a complaint of progressive hair loss of gradual onset of more than 6 months duration were enrolled. In view of the low sensitivity of the Ludwig scale to detect subtle changes in hair density, hair density was rated according to the 5-point visual analog scale that assessed the central part over the scalp (Women’s Alopecia Severity Scale).[6]

Forty male cases clinically diagnosed with AGA were enrolled. Staging of AGA was based on Norwood-Hamilton scale. Men presenting with preserved frontal hair line and thinning over the crown were diagnosed as female pattern hair loss (FPHL) based on the Women’s Alopecia Severity Scale.[6]

Patients with cicatricial alopecia, traction alopecia, trichotillomania or Alopecia Areata were excluded from the study.

Twenty healthy age- and sex-matched volunteers (12 males and 8 females) with no complaint and symptoms of hair thinning or alopecia or history of hair disease were taken as the control group.

Majority of the subjects belonged to the Fitzpatrick skin type IV. Occasionally, the subjects were of types III and V.

### Procedure

The procedure was carried out as per the TrichoScan user manual. All the subjects were asked to wash their hair with a regular shampoo 2 days before the procedure. They were also instructed to avoid using hair oils or hair products.

### Selection of the optimal measurement site for TrichoScan

Representative area of the scalp was prepared for the procedure.

#### Females

The site for both female controls and study cases was same, i.e., mid-scalp region; 1 cm lateral to the midline hair partition between highest points of pinnae of both ears, so that the hairs in close vicinity can be combed over the clipped area.

#### Males

In controls and AGA grade III (with only bitemporal recession, no vertex thinning, no thinning over the crown), the sitewas an area 1 cm behind the frontal hairline in temporal region on either side, whereas in the other cases it was a transitional area of hair loss between normal hair and balding area, i.e., the vertex in cases presenting with vertex thinning or the crown in cases with FPHL.

### Clipping of hair on day 1

The hair exposed through the template was cut close to the scalp surface with a pair of scissors. Thereafter, *Moser-TrichoScan^®^ edition* hair clipper was used to clip the hair evenly and completely to leave a small neat spot where short hair shafts were still visible.

Photographs were taken on day 1 to ensure uniform clipping. However, these photographs were neither stored nor analyzed.

The patients were advised to follow up on the third day for image recording and analysis.

### Dying the hair on day 3

Dying of the hair was done only in individuals with white hairs. The dye product supplied with TrichoScan (Goldwell topchic^®^ black 2 N permanent hair color, Darmstadt, Germany, and Rondo Coiffeur magic color 6% crème-oxyd, Coiffeur, Cologne, Germany) was applied using a wooden spatula after mixing it in a proportion of 1:1 with development cream and left on for 12 minutes before completelyremoving with a spirit swab.

### Recording the images with epiluminescence microscopy

Measurement area was made wet with tap water and the images were recorded with the lens of the digital camera’s (Canon PowerShot A640 10 MP, Keppal Bay Tower, Singapore) optical attachment pressed on the measurement area. This obligatory procedure made sure that no air bubbles were trapped between the scalp and the lens.

### Suitable images for TrichoScan

As an automated image analysis tool, the TrichoScan results strongly depend on the image quality. It was made sure that all the images were optimum with the following features:

- clean and sharp images with no hair dye remnants,
- wet hairs without presence of larger air bubbles,
- no long hairs passing into the measurement site from outside,
- hairs are evenly clipped and dark due to the hair dye and
- hairs have a minimum length of approximately 0.5 mm to allow detection.

### TrichoScan analysis

The recorded photographs were loaded into the TrichoScan software (TrichoScan^®^ Professional Version 3.0; Tricholog GmbH 79117 Freiburg, Germany) which automatically proceeded with the analysis.

### Principle of TrichoScan

**Figure 1:**
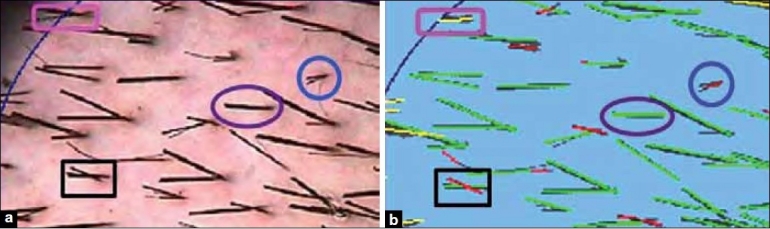
TrichoScan sample picture from TrichoScan manual explaining the detection and color-coding of hair strands. (a) Day 3 image; (b) TrichoScan analysis of the image in Figure 1a. If the hair strand is <0.7 mm in Figure 1a, it will be classified as telogen and coded as red in Figure 1b (blue circle). If the hair strand is >0.7 mm in Figure 1a, it will be classified as anagen and coded as green in Figure 1b (purple oval). Hair strands at the periphery of the analyzed image are coded as yellow (pink rounded rectangle). Moreover, the software claims that even if there are crossing hair strands, they are correctly identified (black rectangle)

TrichoScan is a software program based on statistics and definitions of hair patterns. The software cannot diagnose telogen or anagen hair loss like a histopathologist. However, based on the biologic behavior of those hairs, they can be differentiated by mathematical approximation. By definition, a telogen hair will not grow, whereas anagen hair will (approximately at the rate of 0.3 mm per day). When images are taken 3 days after hair clipping, growing hairs can be differentiated from nongrowing hairs based on the hair length. A default cut-off of 0.7 mm is used to distinguish between growing and nongrowing hair. This cut-off of 0.7 mm can be manually adjusted if needed. TrichoScan identifies nongrowing hairs as telogen hairs (red) and growing hairs as anagen hairs (green) [Figure 1]. The manual of the TrichoScan method states a detection limit of the TrichoScan software as 5 μm hair thickness. Accordingly, it should recognize every hair less than 40 μm in diameter as vellus hair.

### Statistical analysis

The data were tabulated and the groups were compared statistically using Kruskal-Wallis one way analysis of variance on ranks, Mann-Whitney rank sum test and Dunn’s method. These tests were performed with SigmaPlot 11.0. Median values, interquartile ranges (IQR), the 25^th^ and 75^th^ percentiles (25–75%) were used, as the data were not distributed normally.

## RESULTS

Of the 37 women with chronic hair loss, 20 had FPHL stage I, 12 had FPHL stage II and 5 had FPHL stage III. Of the 40 male cases, 16 had AGA grade III, 6 had AGA grade IV, 5 had AGA grade V and the remaining 13 had FPHL stage II.

The mean ± SD ages of the female controls and cases were 27.7 ± 11.4 and 25.3 ± 9.4 years, respectively (*P*=0.253), whereas the mean ages of the male cases and controls were 27.8 ± 5.8 and 23.6 ± 5.4 years, respectively (*P*=0.419).

The demographic characteristics and the total hair densities, telogen hair percentages, vellus hair percentages and the vellus hair density of the female cases and controls are shown in Table 1. Female cases were subgrouped as FPHL I, II and III, and the female controls were compared regarding the hair parameters and the results are summarized in Table 2.

The THD (*n*/cm^2^) between female cases and controls was not significantly different (*P*=0.635). However, THD decreased with increasing stages of FPHL in female cases, the difference being statistically significant only between the groups FPHL stage I and FPHL stage III (*P*=0.003) [Table 2].

**Table 1:** Demographic characteristics and THD, telogen hair percentages, vellus hair percentages and vellus hair density of the female cases and controls

5-point visual analog scale	*n*	Median age (25–75%)	Median THD (*n*/cm^2^) (25–75%)	Median telogen hair percentage (25–75%)	Median vellus hair percentage (25–75%)	Median vellus hair density (*n*/cm^2^) (25–75%)
Control	08	27.5 (21.5–37.5)	291.3 (246–461.7)	32.4 (24.5–36.7)	17.6 (14.6–19.9)	52.8 (38.1–81.4)
FPHL I	20	23.5 (19.5–27.5)	340.2 (298.7–384.7)	30.8 (26.3–44.1)	17.9 (15.2–18.8)	58.1 (50–74.7)
FPHL II	12	24 (20.5–32)	254.4 (215.8–325.9)	34.7 (29.6–39.4)	18.6 (16.4–23.9)	52.9 (44.1–63.9)
FPHL III	05	33 (25–40.5)	212 (107.9–230.4)	29 (24.5–40.8)	17.3 (16.9–18.7)	37.7 (18.2–42.3)

25–75%, range between 25^th^ and the 75^th^ percentile

**Table 2:** THD, telogen hair percentage, vellus hair percentage and vellus hair density in FPHL I, FPHL II and FPHL III and female controls (median)

Females	Controls (*n* = 08)*	FPHL I (*n* = 20)**	FPHL II (*n* = 12)***	FPHL III (n = 05)****	*P* < 0.05
THD (*n*/cm^2^)	291.3	340.2	254.4	212	**/****
Telogen hair %	32.4	30.8	34.7	29	—
Vellus hair %	17.6	17.9	18.6	17.3	—
Vellus hair density (*n*/cm^2^)	52.8	58.1	52.9	37.7	**/****

Tests used- Mann-Whitney rank sum test and Dunn’s method

The median telogen hair percentage of 32.4 in female controls was surprisingly high (the cut-off considered for a normal telogen percentage was taken as 20%, with higher percentages implying effluvium).[7] Whereas, in FPHL stage I, stage II and stage III, the median telogen hair percentages were 30.8, 34.7 and 29, respectively (*P*=0.867) [Table 2].

The median vellus hair percentage was 17.6 in female controls, whereas, in FPHL stage I, stage II and stage III, the median vellus hair percentages were 17.9, 18.6 and 17.3 (*P*=0.569) [Table 2]. Unexpectedly, none of the patients with FPHL stage III, who had obvious thinning of hair, had elevated TrichoScan-analyzed vellus hair percentage (the cut-off considered for a normal small-diameter hair percentage was taken as 20%, with higher percentages implying miniaturization).[8]

Similarly, the hair parameters for male cases and controls were tabulated and compared and have been summarized in Tables 3 and 4.

**Table 3:** Demographic characteristics and THDs, telogen hair percentages, vellus hair percentages and vellus hair density of the male cases and controls

AGA grade	*n*	Median age (25–75%)	Median THD (*n*/cm^2^) (25–75%)	Median telogen hair percentage (25–75%)	Median vellus hair percentage (25–75%)	Median vellus hair density (*n*/cm^2^) (25–75%)
Control	12	23.5 (19.5–26)	255.3 (206.1–305.6)	29.5 (22.6–31.4)	17.1 (16.9–18.3)	42.9 (37.4–51.7)
AGA III	16	23.5 (21.5–26)	257.7 (243.7–271.4)	35.5 (28.9–47.3)	16.3 (15–19.3)	40 (37.5–53.4)
AGA IV	06	25.5 (24–28)	225.8 (218–276.7)	36.9 (32.5–44.3)	18.2 (17.3–21.2)	39.9 (35.1–58.8)
AGA V	05	29 (22.7–38.2)	184.5 (157.8–210.9)	36 (30.8–43.8)	24.8 (19.7–37.6)	56.1 (34.3–65.3)
FPHL II	13	23 (20.8–26.5)	209.7 (181.9–230.8)	28 (28.2–44.4)	17.5 (14.9–20)	40.4 (34.3–65.3)

25–75%, range between 25^th^ and the 75^th^ percentile

**Table 4:** THD, telogen hair percentage, vellus hair percentage and vellus hair density in AGA III, AGA IV, AGA V and FPHL II (males) and male controls (median)

Males	Controls (*n* = 12)*	AGA III (*n* = 16)**	AGA IV (*n* = 06)***	FPHL II (*n* = 13)****	AGA V (*n* = 05)*****	*P* < 0.05
THD (*n*/cm^2^)	255.3	257.7	225.8	209.7	184.5	**/*****
Telogen hair %	29.5	35.5	36.9	28	36	—
Vellus hair %	17.1	16.3	18.2	17.5	24.8	*/*****, **/*****
Vellus hair density (*n*/cm^2^)	42.9	40	39.9	40.4	56.1	—

Tests used were Mann-Whitney rank sum test and Dunn’s method

**Figure 2:**
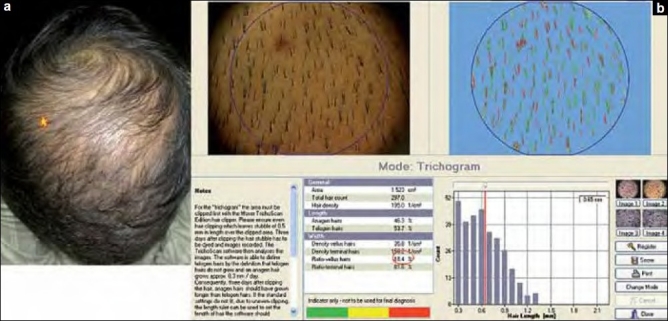
Poor correlation between clinical thinning of hair and TrichoScan analysis of vellus hair percentage. (a) Clinical picture of a male patient with vertex thinning. Star depicting the target area for TrichoScan analysis. (b) TrichoScan analysis of the image taken from the patient in Figure 2a. Red circle highlights the vellus hair percentage reading of 18.4%

Two striking results were noted:

- An elevated telogen hair percentage (>20%) in 17 of 20 controls, i.e., more than 85% of the healthy volunteers had an unexplained abnormal telogen hair percentage. Also, the telogen hair percentages were not significantly different between cases and controls.
- Poor correlation between TrichoScan-analyzed vellus hair percentage and the clinical thinning of hair. This was reflected in the fact that even in the presence of obvious thinning of hair among 17 cases with higher grades of alopecia (AGA IV and V in males and FPHL III in females), only 6 patients had an elevated vellus hair percentage on TrichoScan evaluation, whereas there was no evidence of high levels of vellus hair percentage in the remaining 11 patients. Therefore, in more severe forms of patterned alopecia, vellus hair was not detected in abnormal proportions in 65 % of the patients [Figure 2].

## DISCUSSION

Considering the original claims for accuracy promoting the TrichoScan and a recent study validating the TrichoScan, the above unexpected results of higher telogen counts in controls and lower vellus hair percentages in cases prompted us to investigate further.[4,5] On detailed inspection of photomacrographs of the TrichoScan analyzed images, certain repeated errors in the detection of hairs were underlined. Some of the errors which were repeated in most of the patients are explained below.

**Figure 3:**
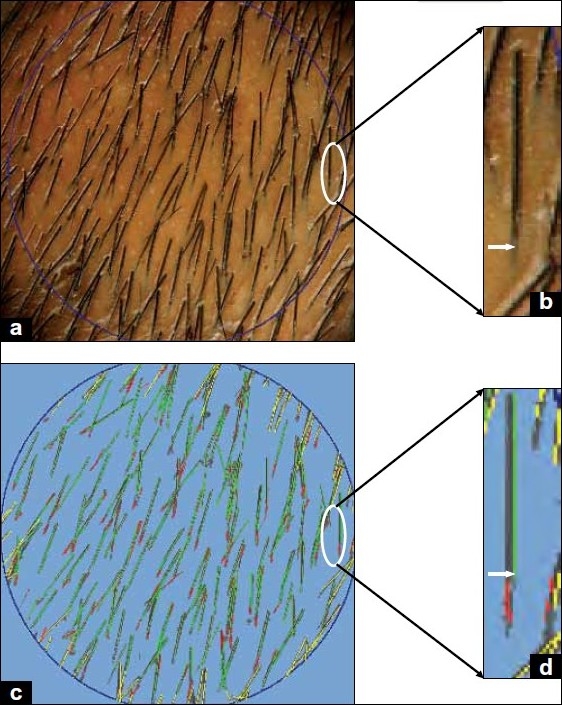
Error in hair detection as it emerges from the scalp (internal scalp fragments of hair). (a) Day 3 image of a patient; (b) magnification of the encircled portion of Figure (a) showing a single strand emerging from the scalp at the scalp ostium (white arrow). (c) TrichoScan analysis of the image in Figure (a); (d) magnification of the encircled portion of Figure (d) showing that the software falsely fragments the single hair strand into two at the point of exit from the scalp surface (white arrow) as a telogen hair (red) beneath the surface of the scalp and an anagen hair (green) above the surface

**Figure 4:**
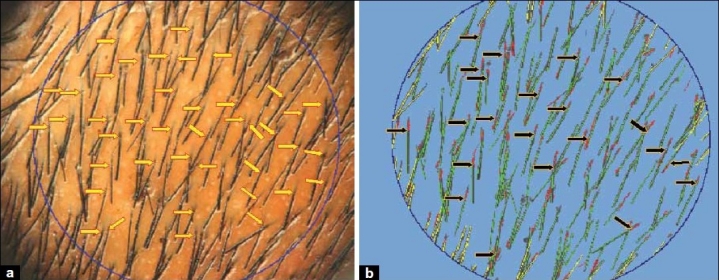
Repetition of errors in hair detection within the same image. (a) Day 3 image of a patient. Yellow arrows mark the points of exit of the hair strands from the scalp ostia. (b) TrichoScan analysis of the image in Figure 4a. Black arrows depict repeated points of exit from the scalp surface where the software falsely analyzed the strand beneath the surface of the scalp as a separate telogen strand

**Figure 5:**
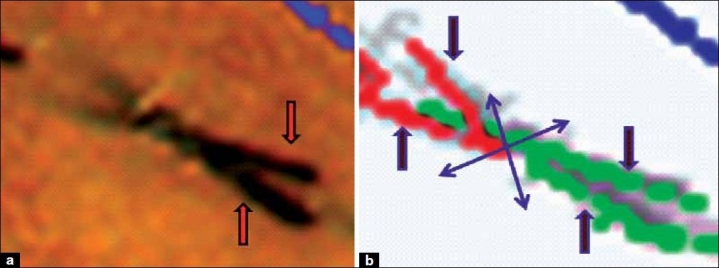
Hair strands crossing each other. (a) Close-up of two hair strands (red arrows) crossing each other. (b) TrichoScan analysis of the hair in Figure 5a. The software fragments the two hair strands at the point of crossing (purple cross) into four strands (arrows): two telogen (red) and two anagen (green)

**Figure 6:**
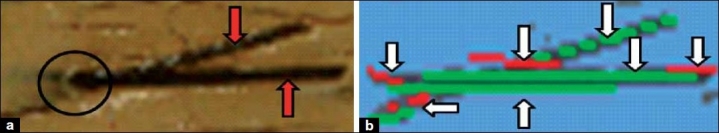
Erroneous fragmentation of hair strands. (a) Close-up of two hair strands (red arrows) emerging from a single ostium (black circle) on the scalp surface. (b) TrichoScan analysis of the hair in Figure 6a. The software falsely fragments the two hair strands into multiple hair (white arrows). These errors are most probably due to the fact that the hair strands in Figure 6a emerge from the same ostium and are in close approximation to each other. Also, the hair strand thickness is not uniform throughout its length

- As the hair strand emerges on the scalp surface from the follicular ostium, the software during analysis breaks a single hair strand into two hair strands – one beneath the surface of the scalp (analyzed as a telogen strand) and one above the surface of the scalp (analyzed as anagen or telogen depending on the length of the strand). This error probably occurs due to the varied pigmentation of the hair strand as it emerges from the scalp ostia [Figure 3].
- Moreover, this was not an occasional finding, but was repeated several number of times even in the same image [Figure 4], thereby falsely elevating the total hair count as well as the telogen hair count.
- Usually, neighboring hair strands are not always oriented parallel to each other and cross each other at several sites. At these sites of crossings, the TrichoScan tends to analyze two crossing hair strands as multiple fragmented hair strands [Figure 5], again falsely elevating the telogen hair counts.
- One to five follicles can emerge from a single scalp ostium (follicular unit). Due to close approximation of hair strands at such places, various errors occur in the detection of hair strands [Figure 6].
- Another situation for error in hair detection is when the hair strand thickness is not uniform throughout its length [Figure 6].

The above findings were in accordance with other studies.

In a study conducted by Van Neste in 2006, which compared performance of TrichoScan with CE-PTG in patients of AGA, it was highlighted that hair fibers escaped the TrichoScan analysis for various reasons that were similar to those of our study. The study also reported that TrichoScan underestimates the hair density and overestimates the telogen ratio.[9]

Similar errors, especially the lack of detection of thinner hair and underestimation of the total hair, have been described by others using the TrichoScan.[9]

Another case-control study by Aktan *et al* in 2007 with females with FPHL (*n*=39), comparing the hair density results of TrichoScan with the results of visual counting using the photomacrographs of the same images concluded that the digital image analysis was found to underestimate mid-scalp hair density by 27.4% in the total group, by 32.6% in controls and by 23.2% in patients with alopecia. In this study, the mean telogen percentage in controls, patients with Ludwig I and Ludwig II were 17.3, 34.4 and 36.3, respectively.[10]

In 2009, in a study where TrichoScan was used to assess patients with FPHL, it was concluded that 50% of patients with thinning had no evidence of miniaturization on TrichoScan vellus hair determination. This finding was further substantiated in our study.[11]

In contrast to the findings of the above studies, a recent study in 2009 validated the TrichoScan technology and concluded that there is an excellent correlation between evaluation of hair parameters using manual identification of hairs and the fully-automated TrichoScan method. However, in this study the TrichoScan values for hair thickness were approximately 10% higher and density values approximately 10% lower than the values obtained by the manual evaluators.

This study was conducted by TrichoScan manufacturer who had contacted an independent Contract Research Organization (CRO) to conduct a validation study. It further proposed that TrichoScan is particularly suitable for clinical studies with treatment comparisons.[5]

Limitations of this study were that only 10 patients with AGA were enrolled and there was no control group. Telogen-anagen percentages were not analyzed and correlation between clinical severity of alopecia and the TrichoScan findings of terminal/vellus hair counts/percentage was not done.

All the original claims made at the launch of TrichoScan are disputed by the errors highlighted in this current study.

To enumerate the drawbacks of TrichoScan are as follows:

- TrichoScan-analyzed telogen hair percentage is falsely elevated.
- TrichoScan-analyzed total hair count and thereby THD measurements are faulty.
- There is no provision to measure hair growth rate.
- Anagen and telogen hair detection is not optimal.
- TrichoScan-analyzed vellus hair percentage does not clinically correlate with thinning of hair.

## CONCLUSION

TrichoScan is promoted as a validated and precise tool for measurement of hair growth parameters. Under certain conditions it may seem suitable for clinical trials evaluating treatment response. We provide evidence that this is an overstatement. This study concludes that TrichoScan-analyzed anagen/telogen hair detection is not optimal. Moreover, there is overestimation of THD and telogen hair percentage and the vellus hair percentage does not correlate with clinical severity of alopecia. The current form of TrichoScan though easy to use is error-prone and awaits refinement. Though computer-assisted image analysis has been reported as a source of potential errors due to the intrinsic complexity of hair growth; this is the first study demonstrating and explaining in detail the actual errors encountered during digital image analysis.

While the impact of computer-assisted image analysis has been largely disappointing, we hope that the next decade will see introduction of improved methods for assessing patients with hair loss, which are both easy to use and devoid of errors.
